# Development and validation of an assessment sheet for all-ceramic crown preparations: A methodological study

**DOI:** 10.34172/joddd.2023.37103

**Published:** 2023-11-11

**Authors:** Ahmed Mhanni, Seham Elsawaay, Abubaker Qutieshat

**Affiliations:** ^1^Department of Prosthodontics, Tripoli Dental Faculty, University of Tripoli, Tripoli, Libya; ^2^Department of Adult Restorative Dentistry, Oman Dental College, Muscat, Oman; ^3^Honorary Researcher, Restorative Dentistry, Dundee Dental Hospital & School, Dundee, UK

**Keywords:** Agreement, Assessment, Dentistry, Pre-clinical skills, Validity

## Abstract

**Background.:**

Dental students learn and practice clinical procedures in clinical skills laboratories. These practices are graded by qualified staff to evaluate the effectiveness of their learning. Valid evaluation requires accuracy and reliability. Although a well-developed checklist for pre-clinical skill evaluation exists in theory, it is challenging to implement in practice. This study was undertaken to develop and evaluate the reliability of an assessment sheet for all-ceramic crown preparations.

**Methods.:**

The study consisted of two phases: the development stage and the judgment-quantification stage. Two examiners evaluated all-ceramic crown preparations made by second-year dental students using the developed assessment sheet to test criterion validity. The final grade was determined based on the number of errors identified using the assessment sheet. The relationship between the negative points and the final grades awarded was determined using Spearman’s correlation. The study calculated the intra- and inter-examiner agreement for two rounds of evaluation, conducted one month apart, using Cohen’s unweighted Kappa test. The study employed the Item-Content Validity Index (I-CVI) to evaluate the content validity for each item and the Scale-Content Validity Index (S-CVI) to assess the content validity of the overall scale used in all-ceramic crown preparation procedures.

**Results.:**

The assessment sheet developed for all-ceramic crown preparations was reliable, with strong content validity and a significant negative correlation between grades assigned and the number of errors observed. The assessment sheet defined up to three levels of performance for each item, providing a consistent and objective approach to evaluation. The linear regression graph successfully determined the maximum number of acceptable errors and established the minimum passing grade. The inter- and intra-examiner agreement for the two assessment rounds was found to be fair to moderate.

**Conclusion.:**

The study showed that the developed assessment sheet for all-ceramic crown preparations is reliable and can provide a consistent and objective approach to evaluation. It can benefit both students and instructors. Further research is recommended to evaluate the impact of the developed assessment sheet on students’ learning outcomes.

## Introduction

 Most dental schools are committed to providing rigorous pre-clinical training as part of their undergraduate program. On the other hand, instructors have struggled to determine how best to assess students’ readiness for the clinical years, during which they will interact with actual patients. Students in pre-clinical training are typically graded and given feedback on their clinical skill performance, which is usually carried out on a set of plastic teeth. This is where the possibility of human error emerges, and objective grading becomes difficult to achieve.^1^

 Academic institutions worldwide use a specific set of assessment checklists to evaluate students’ pre-clinical performance, which are often custom-made to best fit the intended objectives of the course being evaluated. At the same time, instructors would not be able to reliably identify and help the clinically weaker students if they did not have access to a comprehensive assessment approach that could quantify the components of pre-clinical skills and simultaneously incorporate all the tools necessary for effective learning.^2^ To determine whether students have acquired a specific clinical skill, a valid and reliable assessment using an appropriate standard setting should be developed.^3^ There has always been a call for a shift in the evaluation of students’ pre-clinical operative work from the traditional glance-and-grade method to an objective criteria-scoring method that reduces examiner variability. However, this method of scoring cannot be implemented without adequate training and calibration sessions to improve examiner reliability.

 Several studies have been conducted to establish the validity and accuracy of observation as a tool for evaluating the performance of dental students. However, the subjectivity of assessment processes and standards has almost always been a major concern.^4-6^ Faculty assessment inconsistency is a major source of discouragement and the primary motivator for students’ decisions to ‘do just enough to get by.’^4^

 For the assessment instrument to be regarded as valid before examining the reliability of the evaluation tool, it must be able to measure the attributes of the construct under consideration.^7^ Simply put, the validity of an instrument is a critical factor in its application because validity is the extent to which an instrument measures what it is designed to measure.^8^ There are three key components to consider when determining validity for any assessment: content-, criterion-, and construct-related validity.^9^ These validity concepts are used to assess the overall validity of an evaluation procedure.

 The scope of the evaluation procedure may be modified if the examiners determine that the chosen assessment method (e.g., checklist) is irrelevant to the stated learning objectives. Therefore, the assessment method’s ability to adequately cover the subject under evaluation should be ascertained through the content validity viewpoint, which is best delivered by experts in the field.^10,11^ Two steps, called “development” and “judgment-quantification,” make up the process of establishing a checklist’s content validity.^8^ In the “development” stage, a checklist must be created with input and comments from teaching staff and data provided by reputable references on the topic. In the “judgment quantification” stage, the content validity index (CVI) is calculated by having examiners independently rank assessment items and parameters in order of relevance.

 Face validity is provided by a layperson’s acceptance that an assessment tool appears to be sound and robust.^8,11^ Thus, face validity is used to assess understandability, content consistency, and assessment procedure quality. Because face validity cannot be quantified, systematic reviews and pilot studies are frequently used to inform the face validity of the evaluation tool under development.^12,13^

 The skills of dental students are a construct that can be assumed to be greater at the conclusion of the course than they were at the beginning, and this progress would be confirmed by administering an evaluation at the end of the course.^11^ When more capable students learn the skill faster, make fewer mistakes and solve problems better than less capable students, this is a sign that the construct is valid.^14^

 The assessment criteria can be organized in a matrix format, with consistent requirements for each criterion and each criterion extended into written statements explaining various levels of consistency. Examiners can use this evaluation method to determine parameters for each step or feature in a clinical performance task and describe each level of achievement on a scale. All assessment forms would benefit from a clear vocabulary and a formal context, which would support both the student and the clinical examiners.^15-17^

 Using a checklist to determine if the skill’s minimum requirement has been met, in conjunction with a standard setting, should be used to determine whether students have acquired the skill.^18^ Following that, an absolute standard should be carefully developed to justify the pass grade and determine the maximum number of errors that should not be exceeded to receive a pass mark.^3,19,20^

 Examiner experience and bias, grading scale design, student preparation, and instructor expectations all play a role in creating various possible outcomes for an examination. Consistency in grading students is still an area of focus in education reform initiatives worldwide.^21,22^

 Several studies have been published to establish the reliability and accuracy of observation as a tool for evaluating dental student performances.^1^ Intra- and inter-examiner agreement may be assessed by two or more than two examiners.^23^ Percentage agreement, Cohn’s un-weighted kappa, weighted kappa, and intra-class correlation are typical methods for calculating agreement. A percentage agreement may be chosen if the expected by chance is ignored. If there are two examiners, weighted and unweighted kappa tests are used to evaluate intra- and inter-examiner agreement. Both consider the degree of agreement and agreement, which is predicted by chance. The difference is that the unweighted kappa test disregards the degree of disagreement. All disagreement values are assigned a weight of zero for unweighted kappa, while weighted kappa provides different weights for disagreement levels.^23,24^

 The majority of studies on assessment reliability have focused on inter-examiner agreement rather than intra-examiner agreement. As a result, assessment at dental schools has centered on methods to improve calibration among examiners in pre-clinical laboratory courses, with a particular emphasis on techniques to improve consistency among assessors and adjust accordingly to minimize differences between hawk and dove examiners.^25,26^

 This study aimed to develop a valid assessment form for crown preparations in the dental clinical simulation laboratory, demonstrate how to assess intra- and inter-examiner agreement, and finally propose a protocol for evaluating and improving the process of assessing dental clinical simulation skills.

## Methods

 The study was conducted to develop a reliable assessment sheet for all-ceramic crown preparations, given the lack of such a tool within the Dental Faculty where the research was conducted. The study was conducted in two phases: the development stage and the judgment-quantification stage. In the development stage, the assessment sheet was created using three sources: feedback from teaching staff, the grading systems used by examiners, and information from textbooks.^27,28^ The judgment-quantification stage involved two examiners using a four-point scale to determine the relevance of each assessment item. The Item-level Content Validity Index (I-CVI) and the Scale Content Validity Index (S-CVI) were calculated to determine the content validity of each item, and only items ranking 3 (relevant) or 4 (extremely relevant) were retained.

 After creating the assessment sheet items, the criterion validity was tested by two examiners, who evaluated all-ceramic crown preparations made by a cohort of 239 second-year dental students. The examiners were blinded to the identity of the student who prepared each preparation, and they used a visual ranking system and the “fitness-to-category” of preparations to modify the interim grade of each preparation. The final grade was determined based on the number of errors identified using the assessment sheet.

Table 1 shows the interim grade categorization system, while Table 2 lists the criteria used to identify errors. The negative points assigned for each error were used to determine the final grade of each preparation. The relationship between the negative points and the final grades awarded was determined using Spearman’s correlation, with the absolute standard being established using borderline linear regression.

**Table 1:** The initial grading system for all-ceramic tooth preparations at the pre-clinical skills laboratory

**Grade**	**Description **
Grade 1	The student prepared a wrong tooth, unprepared tooth, or prepared a different full crown preparation design
Grade 2	The full crown preparation has not met the standard (not acceptable)
Grade 3	The full crown preparation needs modifications (not acceptable)
Grade 4	The full crown preparation is generally acceptable (acceptable)
Grade 5	The full crown preparation is ideal and meets the standards

**Table 2:** Schematic representation of the assessment sheet items (checklist) for all-ceramic crown preparations (anterior tooth)

**Criteria**	**Performance levels**		
	**Column 1**	**Column 2**	**Column 3**
**Item 1: Incisal surface**			
Incisal reduction	Adequate	Under-prepared	Over-prepared
Incisal inclination	Inclined lingually	Inclination is repairable (e.g., flat)	Not mentioned before (e.g., labially)
Contour of incisal preparation	Yes (followed the tooth surface contour)	Did not follow the surface contour but repairable	Did not follow the surface contour and unrepairable
**Item 2: Axial surface(s)**			
Labial reduction	Adequate for two planes (incisal and gingival plane)	Under-prepared for one or both of planes	Over-prepared for one or both of planes
Contour of labial preparation	followed the tooth surface contour (incisal and gingival plane)	One or two did not follow, but repairable	One or two did not follow and unrepairable
Lingual and cingulum reduction	Adequate for both	Under-prepared for one or both	Over-prepared for one or both
Contour of Lingual and cingulum preparation	Both followed tooth surface contour (lingual or cingulum)	One or two did not follow, but repairable	One or two did not follow and unrepairable
Labio-lingual convergence	Adequate (Convergence)	Improper convergence* but it is repairable	No (destructive shape)** Not repairable
Mesial reduction	Adequate	Under-prepared	Over-prepared
Distal reduction	Adequate	Under-prepared	Over-prepared
Proximal convergence	Adequate (Convergence)	Improper convergence* but it is repairable	No (destructive shape)** Not repairable
Undercuts	No	The undercuts can be repaired	The undercuts are unrepairable
**Item 3: Finish line**			
Shoulder finish line	Yes	Other	
Level of finish line to gingival margin	At gingival (at or above gingival margin by 0.5 mm)	Supra-gingival (above gingival margin more than 1 mm)	Subgingival (under gingival margin)
Depth of finish line	Adequate	Under-prepared for one or more area	Deep/Over-prepared for one or more area
Contour of the finish line all around	Even	Even/Uneven + under-prepared	Even/Uneven + over-prepared
**Item 4: Final preparation**			
Texture of final preparation except for the margin	Adequate (smooth)	Rough (irregular) Sharp edges	
Texture of margin	Smooth and well define	Rough (irregular)	

*Improper convergence, e.g., one wall is tapered, or two walls are parallel. **Destructive shape, e.g., over-prepared, incisal wider than cervical, or too much taper.

 For the agreement part of the study, a cohort of second-year dental students prepared 239 all-ceramic crown preparations, which were evaluated by two academic staff examiners. Intra- and inter-examiner agreement was calculated for two rounds of evaluation, conducted one month apart. The assessment data from both examiners were analyzed using Cohen’s unweighted Kappa test to determine the intra- and inter-examiner agreement, with the grades most agreed upon by each examiner (intra-examiner reliability) and both examiners (inter-examiner reliability) being determined in percentages. The strength of the agreement was interpreted according to Landis and Koch’s criteria.^29^ Data analysis was performed using SPSS 22.0.

## Results

###  Validity assessment

**Figure 1:**
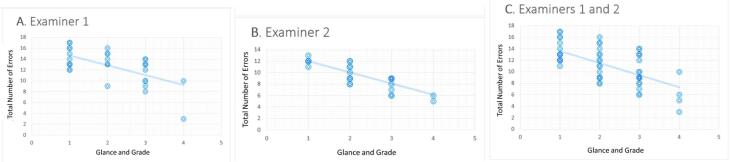
Scatter plot of the grades and the number of errors (negative points) awarded from the ‘assessment sheet’ for two examiners

 The I-CVI and S-CVI were calculated to assess the CVI for items and the CVI for the scale of all-ceramic crown preparations, respectively. The percentage of agreement on relevance for the two examiners was 100%, indicating strong content validity. Figure 1A displays the distribution of negative points awarded by examiner 1 on the assessment sheet for criterion validity. Spearman’s correlation analysis revealed a significant negative correlation between the grades assigned and the number of errors observed by both examiners (*rs* = -0.527, n = 31, *P* < 0.001 for examiner 1; *rs* = -0.781, n = 31, *P* < 0.001 for examiner 2). These findings indicate a strong and very strong relationship between the negative points awarded on the assessment sheet and the previously used ranking scale.

**Figure 2:**
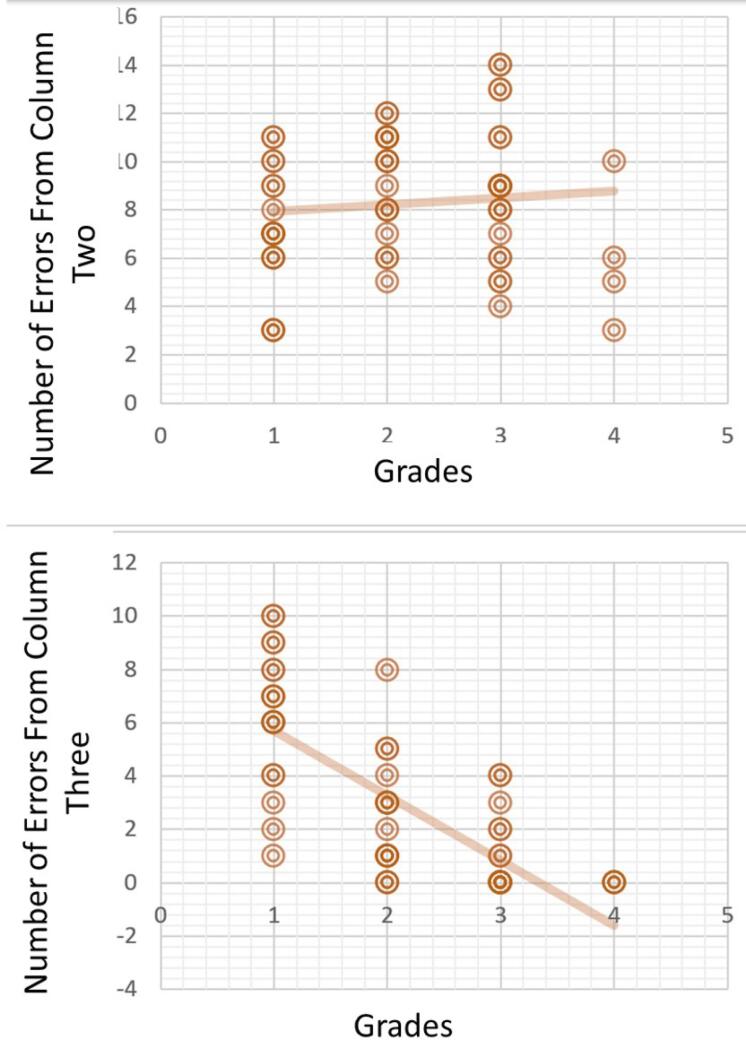
Errors (negative points) are accepted from column 2 (top) and column 3 (bottom) of the assessment sheet for grade 3

 Furthermore, the linear regression graph in Figure 1C was used to determine the maximum number of acceptable errors and establish the minimum passing grade. The maximum negative points in grades 3 and 4 were calculated to determine the definition for each negative point for a passing grade. The assessment sheet defined up to three levels of performance for each item. The first level (column 1) represented an ideal tooth preparation, while the second level (column 2) indicated a tooth preparation that required some adjustments before it could be accepted. The third level (column 3) represented an unacceptable level of preparation that did not meet the standard. The top plot in Figure 2 shows that for grade 3, approximately eight errors from column two were acceptable, while only one error from column three was acceptable. For grade 4, the top plot in Figure 2 shows that nine errors from column two were acceptable, while none of the errors from column three were accepted.

###  Examiner Agreement

**Figure 3:**
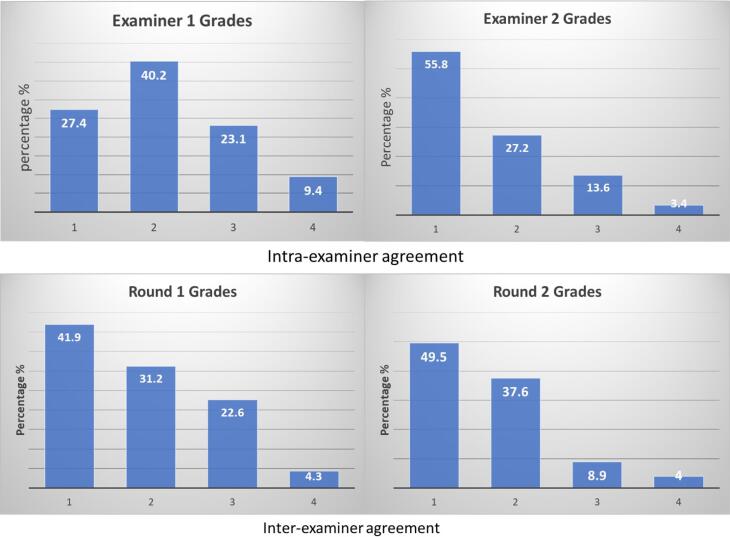
(Top): This diagram illustrates the percentages of the intra-examiner agreement for each grade, as assessed by Examiner 1 (on the left) and Examiner 2 (on the right). (Bottom): This diagram shows the percentages of inter-examiner agreement for each grade in Round 1 (on the left) and Round 2 (on the right)

Table 3 displays the inter- and intra-examiner agreement for the two assessment rounds. The intra-examiner agreement for examiner 1 was fair (kappa value = 0.21‒0.40), while that of examiner 2 was moderate (kappa value = 0.41‒0.60). The agreement for examiner 1 was 49.0%, while for examiner 2, it was 61.5%. The top left graph in Figure 3 shows that grade 2 was more consistent among the rounds (40.2%) for examiner 1, while for examiner 2, the top right graph shows that grade 1 was the more consistent one (55.8%) over two assessment rounds. The inter-examiner agreement was found to be fair for both rounds of assessment, with the agreement between both examiners for round 1 at 38.5% and for round 2 at 42.3%. Figure 3 (bottom) shows that the lowest levels of agreement were demonstrated in grades 3 and 4 in both assessment rounds.

**Table 3:** Cohen’s unweighted kappa agreement of academic staff as examiners of upper central incisor all-ceramic tooth preparations

	**Kappa value**	**Level of agreement**	**Significance (*P*≤0.05)**	**Percentage**
**Intra-examiner**				
Examiner 1	0.301	Fair	0.000	49%
Examiner 2	0.411	Moderate	0.000	61.5%
**Inter-examiner**				
Examiner 1	0.266	Fair	0.000	38.5%
Examiner 2	0.249	Fair	0.000	42.3%

## Discussion

 Preparing a dental crown is a complex task that requires precision, repeatability, and the ability to conceptualize in three dimensions. As a result, dental students must practice extensively before becoming proficient in this psychomotor skill. However, evaluation procedures can be arbitrary, negatively affecting students’ confidence and performance. Therefore, the assessment method used should be both valid and reliable.

 The dental school where this work was conducted employs the glance-and-grade assessment method to evaluate students’ performance. Despite its low cost, this assessment method does not demonstrate examiner reliability.^18^ Assessment methods, such as computer-assisted learning and computer-assisted simulation systems, are currently being developed to objectively evaluate dental preparations.^30,31^ Although they provide students with consistent, accurate, and objective assessments, they are costly, time-consuming to design and require training to provide reliable results.^32,33^ These newer and more sophisticated methods for evaluating student performance still require checklist elements and tooth preparation measurements to function properly and provide meaningful evaluations of student work. Although checklist assessment is a low-cost and widely used method in dental education, it has rarely been validated.^18^ Therefore, whether the checklist will be used as-is or as a part of an automated assessment system, it should always be validated.

 This study demonstrated a slight but palpable increase in agreement percentage when a validated checklist was used. It is reasonable to believe that additional training on the checklist’s use will lead to better agreement in the long run. Additionally, due to the inherently subjective nature of esthetic judgment in dental work,^34^ it is advised that dental examiners focus more on the “objective” aspects of clinical performance, such as final preparation shape and dimensions, and pay less attention to the “subjective” areas. By emphasizing the mechanical and scientific aspects, examiners can enhance agreement and reduce the potential influence of non-essential artistic touch on their judgment. School boards should encourage examiners to carefully observe “experts” during assessments, as these experts can act as “models” to showcase the interaction of subjective and objective aspects of performance.

 It is worth noting that using a validated checklist is not a panacea for all assessment challenges in dental education. While a validated checklist can provide a more objective evaluation, it should not be the only tool to assess clinical skills. Clinical skills involve not only technical proficiency but also patient communication, clinical decision-making, and professionalism.^35^ Evaluating these aspects of clinical skills requires a more comprehensive approach that incorporates multiple assessment methods, including direct observation and self-assessment. Bilan et al^36^ underline that a successful shift to competency-based evaluations hinges on robust infrastructural support and faculty empowerment, which, in our context, pertains to training, standardization of assessment procedures, and calibration exercises to ensure examiner consistency. Furthermore, they highlight the essential role of faculty feedback and recognition for innovations in educational assessment methods, indicating that such institutional support can potentially enhance the successful implementation and effectiveness of a validated checklist. While important, using a validated checklist does not necessarily guarantee that all examiners will agree on the assessment. Therefore, continued engagement with, training on, and refining the checklist are key to minimizing examiner variability and improving agreement.^37^

 This paper presented a conceptual guideline for developing a valid and reliable checklist as a first step toward improving clinical assessment evaluation. The research also yielded implementation recommendations that should be implemented when considering a goal, such as reducing the subjectivity of student clinical work evaluation. These recommendations are relevant and useful for any institution looking to improve the reliability and validity of its clinical skills assessments.

 The first step in implementing a reliable checklist for assessing dental students’ crown preparation skills is to evaluate the existing agreement between faculty members within a single department or discipline. This can be done by having multiple examiners assess students’ performance using various evaluation methods, including eyeballing, specific measurement tools, and/or a checklist sheet. The results should then be analyzed to determine intra- and inter-examiner agreement based on the grades awarded.

 To further improve the consistency of examiner comments and grades, it is important to evaluate the examiners’ ability to assess students’ work. This can be done by allowing examiners to evaluate students’ performance using the above-mentioned evaluation methods. The results should be compared to determine the correlation between grades and negative points awarded by the examiners using the checklist sheet and to determine intra- and inter-examiner consistency.

 Once the initial evaluation is complete, the next step is to determine whether or not the grades assigned based on the checklist sheet accurately reflect the assessment’s intended outcomes. This can be done by taking a dual perspective on the checklist sheet and evaluating it objectively (using specific tools and/or software) and subjectively (using the examiners’ expertise and intuition). The former should be compared to the body of evidence and recommendations in the current literature for the skill under consideration, while the latter can be accomplished by restricting the examiners’ valuation to a binary response, such as yes/no.

 The pass and fail scores can then be calculated using Albino and collegues’^38^ faculty calibration recommendations, which is usually followed by comparing these scores to those given by examiners via agreement tests (e.g., kappa test or agreement percentage). This process will ensure that the checklist is reliable and valid in assessing the intended outcomes.

 The final step is the development stage, which involves identifying the most reliable assessment tools and refining the preliminary checklist to ensure that the evaluation process is tailored to its intended purpose. This step typically follows the evaluation phase and entails comparing the grades awarded during the evaluation of the examiners’ ability to assess with those awarded from the checklist sheet’s objective and subjective evaluations. The results would confirm the improvement of the preliminary checklist, as well as information gleaned from current literature and/or other recommendations proposed by the examiners.

 The new checklist can then be used to determine intra- and inter-examiner agreement based on grades and negative points awarded. The correlation between grades and negative points and the consistency and agreement among examiners can be computed. By following these recommendations, dental schools can implement a reliable checklist for assessing dental students’ crown preparation skills, ensuring consistent and accurate evaluations that will help students develop their skills and prepare for successful careers in dentistry.

 While the recommendations presented in this paper are intended to provide a comprehensive framework for developing and implementing a dental clinical skills assessment checklist, it is important to note that the study had several limitations. First, the recommendations have not been empirically tested in a large-scale study. While the recommendations draw on existing literature and expert opinion, they might not be as effective in practice as they appear on paper. Therefore, further research is necessary to evaluate the effectiveness of the recommendations in real-world settings. Second, the recommendations are specific to assessing crown preparation skills in dental simulation laboratories. While they may hold value for similar technical procedures, their applicability to other types of clinical skills assessments, especially those involving complex interpersonal interactions or decision-making processes, may be limited. Therefore, further research is required to explore the adaptation and validation of this assessment framework for other clinical skills, considering the unique aspects and challenges inherent to each skill set. Third, the recommendations assume a certain level of expertise and resources on the part of the instructors and institutions implementing the assessment. Therefore, it may not be feasible for all institutions or instructors to follow the recommendations as outlined. Fourth, while our study offers valuable insights into developing and validating an assessment sheet for all-ceramic crown preparations, it was conducted within a single dental faculty. As such, the influence of individual differences among instructors and students within our institution may affect our findings’ generalizability. Future studies should aim to replicate our research across different dental faculties to assess the cross-institutional reliability and validity of the assessment sheet. Finally, the recommendations may be subject to bias or errors due to the subjective nature of clinical skills assessment. Instructors need to remain vigilant and objective in their evaluation of students and to continually reevaluate and refine the assessment process to minimize the impact of such biases or errors.

## Conclusion

 In conclusion, the recommendations provided in this paper offer a clear and effective strategy for implementing a dental clinical skills assessment checklist in a clinical simulation laboratory. By following the suggested steps, tutors and course instructors can objectively evaluate students’ dental clinical simulation skills, including crown preparation, and identify those who may require additional instructions or support.

 Using a standardized assessment tool, like the checklist, not only provides a more reliable and consistent means of evaluating students’ performance but also allows for more targeted instructions and feedback. With this approach, instructors can focus on addressing specific areas where students need improvements and track progress over time.

 Overall, implementing a dental clinical skills assessment checklist can improve the quality of dental education and training, leading to better patient outcomes and improved professional practice. By adopting the recommendations outlined in this paper, dental educators and practitioners can help ensure that students are adequately prepared for the clinical challenges they will face in their future careers.

## Competing Interests

 There are no conflicts of interest.

## Ethical Approval

 Not applicable.
